# Editorial: Ubiquitination in tumor pathogenesis and progression and its therapeutic potential

**DOI:** 10.3389/fimmu.2026.1786943

**Published:** 2026-01-21

**Authors:** Asif Amin Dar

**Affiliations:** Department of Neurology, The Children’s Hospital of Philadelphia, Philadelphia, PA, United States

**Keywords:** cancer pathogenesis, E3 ligases, immune modulation, targeted protein degradation, tumor immune microenvironment, ubiquitination

Ubiquitination is a fundamental post-translational modification that regulates protein stability, signaling intensity, and cellular identity (1–3). Orchestrated through a hierarchical enzymatic cascade involving E1 activating enzymes, E2 conjugating enzymes, and highly selective E3 ubiquitin ligases, ubiquitin modification governs diverse processes including DNA damage repair (4), cell cycle progression (5, 6), immune signaling (7, 8), and metabolic homeostasis regulation (9–11). Rather than acting as a passive quality-control mechanism, ubiquitination functions as a dynamic regulatory system that enables rapid cellular adaptation to environmental and physiological cues.

In cancer, this system is frequently co-opted (12). Tumor cells exploit ubiquitin-dependent pathways to degrade tumor suppressors, stabilize oncogenic drivers, reprogram metabolism, and evade immune surveillance (7, 10, 13, 14). Genetic alterations and functional dysregulation of E3 ligases, deubiquitinases (DUBs), and proteasome components are now recognized as recurrent events across solid tumors and hematologic malignancies. These insights have repositioned ubiquitination from a downstream consequence of transformation to a central driver of tumor initiation, progression, and therapeutic resistance (13).

This Research Topic brings together mechanistic and translational studies that move beyond descriptive cataloging of ubiquitin pathway components to interrogate how ubiquitin-dependent regulation actively shapes malignant behavior. Collectively, the contributions underscore both the therapeutic promise of targeting ubiquitination and the need for precision in doing so.

## Ubiquitin-dependent mechanisms sustaining malignancy

Several studies in this Research Topic provide mechanistic clarity into how tumors leverage ubiquitin circuitry to maintain survival and proliferative advantage.

Liu et al., identify BCL2-associated athanogene 2 (BAG2) as a critical mediator of apoptotic resistance in gastric cancer. By inhibiting CHIP-mediated ubiquitination, BAG2 stabilizes HSP70 and suppresses apoptosis. Importantly, the authors demonstrate that pharmacologic disruption of the BAG2–CHIP interaction restores apoptotic signaling, highlighting protein–protein interactions within ubiquitin pathways as therapeutically tractable vulnerabilities. This study exemplifies an emerging principle in the field: effective targeting of ubiquitination may require disrupting specific oncogenic dependencies rather than globally inhibiting the ubiquitin–proteasome system.

In hepatocellular carcinoma, Peng et al., reveal an unexpected oncogenic role for PSMD12, a non-ATPase subunit of the 26S proteasome. PSMD12 promotes tumor growth by stabilizing CDK1, thereby accelerating G2/M cell-cycle progression. These findings challenge the notion that proteasome components act solely through global proteolysis and instead highlight subunit-specific proteasome functions that can selectively drive malignancy.

Li et al., identified ubiquitination-driven molecular subtypes in thyroid cancer and defines a four-gene ubiquitination signature with strong prognostic value. The close association of this signature with immune infiltration, immune checkpoint expression, and predicted immunotherapy responsiveness underscores the translational potential of ubiquitination-based biomarkers for patient stratification and therapeutic decision-making.

Hematologic malignancies are addressed by Xian et al., who identify ubiquitination-related gene signatures that stratify patient risk in acute lymphoblastic leukemia. Notably, the E3 ligase FBXO8 emerges as a protective factor; its loss enhances leukemic proliferation while promoting an immunosuppressive microenvironment characterized by regulatory T cells and M2 macrophages. This work reinforces a critical concept: ubiquitin dysregulation often couples tumor-intrinsic growth programs with immune evasion, rather than acting in isolation within cancer cells.

Extending the scope beyond signaling and cell-cycle control, Zhang et al., review how ubiquitination regulates lipid metabolic reprogramming in pediatric solid tumors. By controlling enzymes involved in cholesterol biosynthesis, fatty-acid uptake, and β-oxidation, ubiquitin pathways support tumor growth and immune escape. This synthesis highlights metabolism as a key downstream effector of ubiquitin signaling and identifies metabolic dependencies as promising therapeutic entry points, particularly in pediatric cancers where treatment options remain limited.

## Targeting ubiquitination in cancer: context, complexity, and consequence

Despite growing enthusiasm for ubiquitin-targeted therapies (15), key challenges persist. Ubiquitination is highly context-dependent, with the same E3 ligase or deubiquitinase exerting divergent effects across cell types, immune states, and disease stages, complicating therapeutic prediction and biomarker interpretation. Beyond tumor cell–intrinsic functions, ubiquitin ligases and DUBs regulate immune cell development, cytokine receptor turnover, activation thresholds, and exhaustion states (8, 16–19). These pathways are therefore central not only to oncogenesis but also shape the tumor immune microenvironment.

This functional duality creates a core therapeutic dilemma. While selective modulation of ubiquitin pathways can suppress malignant growth or pathogenic inflammation, indiscriminate targeting risks disrupting protective immunity and immune homeostasis. Broad proteasome inhibition, despite efficacy in select hematologic malignancies (20), exemplifies these limitation through systemic toxicity and immune consequences.

Collectively, the studies in this Research Topic underscore a unifying principle: context matters, and successful ubiquitin-targeted therapies must prioritize cellular specificity, immune awareness, and precise substrate control.

## Future directions

To fully harness the therapeutic potential of ubiquitination in cancer, several priorities emerge:

1. Context-specific functional mapping: Systematic annotation of E3 ligases and DUBs across tumor and immune compartments—using CRISPR screening, proteomics, and single-cell approaches—is essential to distinguish oncogenic dependencies from homeostatic functions.
2. Selective ubiquitin modulation: Emerging technologies such as proteolysis-targeting chimeras (PROTACs) and molecular glues offer opportunities to exploit specific ubiquitin ligase–substrate interactions rather than globally suppressing proteostasis.
3. Rational combination therapies: Integrating ubiquitin-targeted agents with immunotherapies, metabolic inhibitors, or conventional treatments may overcome resistance mechanisms while minimizing toxicity.
4. Biomarker-driven patient stratification: Ubiquitination signatures integrated with immune and metabolic profiling may enable precision treatment strategies and improve patient selection.

## Conclusion

The contributions to this Research Topic establish ubiquitination as an active and central regulator of tumor pathogenesis rather than a passive protein-quality-control system. By coordinating oncogenic signaling, metabolic adaptation, and immune modulation, ubiquitin pathways occupy a critical nexus in cancer biology.

At the same time, these studies highlight a cautionary principle: the therapeutic power of ubiquitination lies in its specificity. Future success will depend on moving away from broad pathway inhibition toward precise, context-aware manipulation of ubiquitin circuits. Continued integration of cancer biology, immunology, and translational research will be essential to convert these mechanistic insights into safe and durable cancer therapies.
